# Anthropometric multicompartmental model to predict body composition In Brazilian girls

**DOI:** 10.1186/s13102-017-0088-7

**Published:** 2017-12-21

**Authors:** Dalmo Machado, Analiza Silva, Luis Gobbo, Paula Elias, Francisco J. A. de Paula, Nilo Ramos

**Affiliations:** 1 School of Physical Education and Sport of Ribeirao Preto, University of Sao Paulo, Bandeirantes Ave. 3900, Monte Alegre, Ribeirão Preto, SP 14040-900 Brazil; 2 Exercise and Health Laboratory, CIPER, Faculdade de Motricidade Humana, Universidade de Lisboa, Lisboa, Portugal; 3 Department of Physical Education, Universidade Estadual Paulista, Presidente Prudente, Ribeirao Preto, Brazil; 4 Department of Internal Medicine, Ribeirao Preto Medical School, University of Sao Paulo, Ribeirao Preto, SP Brazil; 5 Department of Graduate and Specialty Studies, Coastal Carolina University, Conway, SC USA

**Keywords:** Multicompartmental analysis, Children, Adolescents, Equation, DXA

## Abstract

**Background:**

Anthropometric models remain appropriate alternatives to estimate body composition of peripubertal populations. However, these traditional models do not consider other body components that undergo major changes during peripubertal growth spurt, with restrictions to a multicompartimental approach as a quantitative growth. DXA has great potential to determine pediatric body composition in more than one component (3-C), but has limited use in field settings. Thus, the aim of this study was to propose and validate an anthropometric model for simultaneous estimation of lean soft tissue (LST), bone mineral content (BMC) and fat mass (FM) in healthy girls, from a multivariate approach of densitometric technique, as the criterion method.

**Methods:**

A sample of 84 Brazilian girls (7-17 years) was defined by chronological age and maturity offset. Whole total and regional DXA body scan were performed and, the components were defined (LST, BMC and FM) and considered as dependent variables. Twenty-one anthropometric measures were recorded as independent variables. From a multivariate regression, an anthropometric multicompartmental model was obtained.

**Results:**

It was possible to predict DXA body components with only four predictive measurements: body weight (BW); supra-iliac skinfold (SiSk); horizontal abdominal skinfold (HaSk) and contracted arm circumference (CaCi) with high coefficients of determination and low estimation errors (**LST** = 0.6662657 BW - 0. 2157279 SiSk - 0.2069373 HaSk + 0.3411678 CaCi - 1.8504187; **BMC** = 0.0222185 BW - 0.1001097 SiSk - 0.0064539 HaSk - 0.0084785 CaCi + 0.3733974 and **FM** = 0.3645630 BW + 0.1000325 SiSk - 0.2888978 HaSk - 0.4752146 CaCi + 2.8461916). The cross-validation was confirmed through the sum of squares of residuals (PRESS) method, presenting accurate coefficients (Q^2^_PRESS_ from 0.81 to 0.93) and reduced error reliability (S_PRESS_ from 0.01 to 0.30).

**Conclusions:**

When sophisticated instruments are not available, this model provides valid estimates of multicompartmental body composition of girls in healthy Brazilian pediatric populations.

## Background

Assessment and monitoring of body composition in children and adolescents have great significance when there is the need to: a) study the prevalence of pediatric obesity, b) improve gender screening of body composition, c) track body composition from healthy childhood to adulthood, d) to assess FM changes over time in a given population [1], e) evaluate sport potential of young people, f) monitor training process, and g) prior knowledge of their physical characteristics [2].

Anthropometric based equations continue to be adequate alternatives to determine the body composition of pediatric populations. However, body composition assessment in children is not easy to measure, since the relationship between body components during growth is not constant as in adults [2]. The progress in the study of the quantitative human two-compartment model (2-C) comprising FM and fat-free mass (FFM) to 3-C template (water, fat and residual mass) and 4-C with the estimation of other components in addition to FM, total-body water (TBW), minerals and protein [3], has provided new ways for approaching the traditional body composition, especially when it involves peripubertal people.

Several pediatric anthropometric equations were developed using a model of 2-C from the hydrostatic weighing [4, 5] and other densitometric techniques. However, this approach is based on assumptions of stable relationship for FFM density (1.1 g/cm^3^) and FFM hydration (73.2%). These values are stable in adults, but vary substantially during growth [6, 7]. In fact, from birth to adolescence bone mineral and protein increase whereas TBW decreases thus raising FFM density until reaching the adult value when the chemical maturity profile is reached [6].

Therefore, body composition models have a multicompartmental approach, as the reference method, which accounts for the variability of the main FFM components due to age and maturational changes, resulting in more valid equations [1]. Even using 4-C models, several anthropometric models have been developed to estimate one body component, usually FM for pediatric populations. However, it could be possible to estimate other components such as water, protein, and mineral. In addition to using DXA as the reference method to develop anthropometric models, lean-soft tissue (LST), FM, and bone mineral content (BMC) could also be determined by using a multivariate regression model. When conceived in a multivariate pattern, considering appropriately all important factors, the likelihood to create robust models is attainable, increasing the predictive capacity and reducing errors of estimation [8–10].

Currently, DXA is recognized to have a great potential to determine body composition in pediatric studies [7], due to its ability to provide more than one component (3-C approach). However, the exposition to ionizing radiation, the equipment cost along with the lack of feasibility for large-scale use limits its applicability in field settings (home, school environments, and sport clubs). Consequently, simple solutions to estimate body composition in children from anthropometric techniques including skin folds, bone breadth and circumferences have been widely used and are preferred in different contexts [1]. These alternatives are more convenient due to their low costs, they require low level of personnel training, they are minimally invasive and have good scientific credibility [11].

Although conventional anthropometric methods are scientifically accepted, they do not distinguish bone from fat or muscles, a desirable fractionation of the body composition, in youth sports setting or clinical health. With the exception of the anthropometric models developed in our laboratory to determine LST, FM and BMC of boys [10–12], no proposal for girls was found able to estimate these 3 components, by somatic body measurements.

## Methods

The aim of this study was to develop and validate a multicomponent anthropometric model to simultaneously estimate LST, BMC and FM in girls, from densitometry as a criterion method. This descriptive study design was conducted with an intentional sample from elementary school and swimming sporting centers, a social project for local children and adolescents.

### Study population

The study followed a cross-sectional design and a sample of 84 girls between seven and 17 years recruited from a sports center (n = 69) and elementary schools (n = 15). The girls were healthy with no medical condition, without body parts amputated, no use of drugs or not under medical treatment that could affect metabolism, appetite or growth. All participants presented maturation status below the 5 Tanner stage, and body mass index (BMI) below pediatric obesity [13], except 7 cases slightly above each age limit.

### Study protocol

All evaluations were performed at the Imaging Center of the University Hospital, Ribeirao Preto Medical School/University of São Paulo, after an overnight fasting, in a single session; the same technicians performed all exams. Before the measurements, the subjects were asked to empty their bladders. A total-body DXA scan was performed according to manufacturer's guidelines. The anthropometric measures were taken according to the literature guidelines (15, 16), whose procedures are summarized below.

### The dependent variables

Whole and regional body composition was estimated with a DXA Scanner Hologic® (Discovery CI/WI, software version 11.2, Bedford, MA). The Lean Soft Tissue (LST, kg), Bone Mineral Content (BMC, kg) and Fat Mass (FM, kg) were determined (3-C) and considered as dependent variables.

### The independent variables

The subject’s body mass and height were measured with a digital scale (Welmy®, W 300 A, Santa Barbara d’Oeste, SP) and a hall fixed stadiometer (Physical Therapy Stadiometer - Terrazul®, Sao Paulo, SP), respectively. The skinfolds (n = 10), circumference (n = 10) and widths (n = 9), were measured by conventional procedures in the literature [14, 15] using Prime® equipment. Chronological age groups in decimal value were adjusted to the nearest integer [16]. To ensure the reliability of the results used in the models, intra evaluator technical error of measurement, absolute (TEM) and relative (TEM%), were determined in subsequent days. Thirteen subjects were evaluated in duplicates for all variables used in this study, when the results were always within the expected tolerance limits [14] as shown below in Table 1.

**Table 1:** Anthropometrics and DXA body composition measures in girls (n = 84), including absolute and relative TEM.

	Min-Max	Mean	CI 95%	SD	TEM	TEM%
Milesimal age (years)	7 – 17	11.64	11.11 to 12.16	2.42	-	-
PHV (years)	-6.05 – 1.27	-3.16	-3.48 to -2.83	1.51	-	-
*DXA measures*						
Lean soft tissue (kg)	12.55 – 53.39	29.28	27.53 to 31.04	8.08	0.06	0.15
Bone mineral content (kg)	0.74 – 2.55	1.44	1.35 to 1.53	0.43	0.01	0.03
Fat mass (kg)	5.56 – 46.05	16.51	14.98 to 18.05	7.08	0.22	1.42
*Anthropometrics*						
Stature (kg)	116.0 – 178.0	148.00	144.93 to 151.08	14.16	0.17	0.11
Body mass (kg)	19.4 – 101.0	46.84	43.78 to 49.89	14.06	0.27	0.29
BMI (kg/m^2^)	14.3 – 38.0	20.98	19.97 to 21.99	4.65	-	-
Seat height (cm)	70.0 – 105.0	86.38	84.78 to 87.97	7.35	0.26	0.30
Triceps skinfold (mm)	4.3 – 35.2	15.74	14.22 to 17.26	6.99	0.12	1.09
Subscapular skinfold (mm)	2.0 – 38.0	12.66	11.04 to 14.27	7.43	0.04	0.28
Suprailiac skinfold (mm)	2.5 – 54.0	17.56	15.32 to 19.80	10.34	0.35	2.27
Horizontal abdominal Skinfold (mm)	5.8 – 60.0	21.57	19.43 to 23.72	9.89	0.82	4.20
Thigh skinfold (mm)	9.8 – 58.0	23.76	21.78 to 25.74	9.13	0.63	3.39
Media calf skinfold (mm)	4.1 – 33.0	16.70	15.25 to 18.15	6.69	0.23	1.88
Relaxed arm circumference (cm)	16.0 – 36.0	23.03	22.20 to 23.20	3.83	0.31	1.35
Contracted arm circumference (cm)	16.7 – 36.8	23.97	23.18 to 24.77	3.67	0.08	0.30
Waist circumference (cm)	48.0 – 106.3	64.58	62.68 to 66.48	8.75	0.37	0.56
Abdominal circumference (cm)	52.0 – 117.0	70.45	68.21 to 72.69	10.32	0.48	0.67
Hip circumference (cm)	59.0 – 118.5	83.25	80.70 to 85.80	11.74	1.33	1.50
Thigh circumference (cm)	30.0 – 68.0	46.10	30.00 to 68.00	7.17	0.70	1.47
Calf circumference (cm)	21.0 – 42.0	30.67	29.77 to 31.57	4.15	0.11	0.33
Bi-acromial breadth (cm)	17.0 – 55.7	32.14	31.19 to 33.09	4.38	0.22	0.61
Bi-iliac breadth (cm)	17.0 – 33.6	24.00	23.37 to 24.63	2.92	0.22	0.86
Biepicondylar humerus breadth (cm)	4.1 – 6.9	5.55	5.43 to 5.67	0.57	0.01	0.18
Biepicondylar femur breadth (cm)	4.1 – 11.5	8.32	8.12 to 8.53	0.94	0.02	0.17

*SD*, Standard deviation, *CI* Confidence interval, *Min-Max* minimum – maximum, *TEM* absolute technical error of measurement, *TEM%* relative technical error of measurement, *DXA* Dual-energy X-ray absorptiometry, *PHV* years for peak height velocity

Their maturity offset was determined by gender-specific regression to predict the years for Peak Height Velocity (PHV) according to the following equation [17] for girls:

$$ {\displaystyle \begin{array}{c}\mathrm{PHV}=\hbox{-} 9.376+0.0001882\ \left(\mathrm{Lh}\;\mathrm{x}\;\mathrm{Sh}\right)+0.0022\ \left(\mathrm{A}\;\mathrm{x}\;\mathrm{Lh}\right)+0.005841\ \left(\mathrm{A}\;\mathrm{x}\;\mathrm{Sh}\right)\hbox{-} 0.002658\ \left(\mathrm{A}\;\mathrm{x}\;\mathrm{Wt}\right)+0.07693\\ {}\left(\left(\mathrm{Wt}/\mathrm{Ht}\right)\ \mathrm{x}\ 100\right)\end{array}} $$

*Where: Lh stands for legs height (cm), Sh stands for seating height (cm), A stands for age (years), Wt stands for body mass (kg), and Ht stands for height (cm).*

### Statistical analysis

Interaction terms for ethnicity (Brazilians are highly intermingled) by anthropometric variables and sports activities (initial swimming practitioners) by anthropometric variables were tested and a nonsignificant differences was found (*p* = 0.238), allowing the use of the entire sample for subsequent analysis.

A sampling design was planned to set the desired maximum error (*ε*) with some degree of confidence (*Z*_*y*_) from the prior knowledge of the population variability (*σ* ^2^) [18]. In this case, a multiethnic classic study values [19] describing girls body composition of different populations was used as reference values. The highest variability was observed in FM estimated by DXA, for all that ages (6-18 years), similarly to the sample ages of this study (7-17 years). From the estimation of the predetermined error (between 2.25 and 2.0%) and confidence interval (0.90 to 0.95), the ideal n for our study was defined by the equation [18]:

$$ n={\left[\frac{Z_yS}{\varepsilon}\right]}^2 $$

The minimum sample values (*n* = 74), setting for *ε* = 2.25 and γ = 0.95 and the estimated value of FM to σ = 9.84 Kg was overcome by our sample (n = 84), from the standard deviation (*s*) obtained of that (Hispanic group) reference sample [19].

### Multicompartmental anthropometric equation development

As earlier procedures [11] multivariate regression model (_**n**_ **Y**_**m**_=_**n**_ **X**_(**r** + **1**) (**r** + **1**)_ **β**_**m**_+_**n**_ **ε**_**m**_) by diagonal mutual analysis, parameter estimation and the least squares errors method was used [8] by R-Free Software. Previously, a criteria for selection and reduction of independent variables was conducted from the following steps: a) principal component analysis and model adequacy (Kaiser-Meyer-Olkin) and Sphericity test (Bartlett); b) univariate regression (stepwise) to determine all common independent variables for each dependent variable (LST, BMC and FM), with significance less than 5%; c) multivariate analysis to estimate the parameters and Pillai approximation method for displaying possible variables exclusions; e) testing of remaining model (enter - univariate method), with estimated values of VIF (<10.0) and multicollinearity (L <1000) maximum permitted; f) adjustments by Pillai approach to test the F values; g) set the number of independent variables, with high coefficients of determination (r ≈ 0.90). Then β parameters (multivariate) were determined, with the proposition of equations and residual distribution for each dependent variable. A similar procedure was earlier conducted in boys [10].

The PRESS statistic was used to measure the efficiency of a predictive equation, when applied to independent samples [20], that is, when used to estimate within the same sample. The process may be understood as design efficiency in estimating the actual parameters by a virtual simulation. In the case of this study, the error was determined by the result of observed Y - Y ' (estimated). For validation: a) the correlation coefficients were estimated between predicted and measured values, and b) cross-validation by PRESS method and coefficients of determination (Q^2^_PRESS_) and error (S_PRESS_) was conducted for each dependent variable, according to the Johnson and Wichern [8] recommendations.

Other statistical procedures included descriptive analysis with calculation of mean and standard deviation and confidence interval (95%) and coefficient of variation, used to express milesimal age, DXA and anthropometric dimensions of girl’s body components.

## Results

Table 1 provides anthropometric and body composition measures, including range (minimum – maximum), the confidence interval (95% CI), and absolute and relative TEM of the measures expressing the main characteristics of the study sample.

The Kaiser-Meyer-Olkin test (0.882) was used as a measure of adequacy of the sample (*X* ^*2*^ = 2961.11) with *p* < 0.001, indicating suitability of the method in the treatment of the data. The commonalities test ranged from 0.689 to 0.927 for the 22 variables entered into the analysis. The matrix indicated coefficients of correlation from weak (*r* = 0.20) to very high (*r* = 0.95) between the 21 independent variables (size measures, skinfolds, circumferences, bone breadths) and the dependent variables (data not shown). To reduce variables of the model, univariate regression was performed (stepwise) in order to select the common dependent variable to the three components (LST, BMC and FM) since common selection occurred at least twice, maintaining the highest possible coefficients.

The high correlation between the independent variables shows the existence of multicollinearity, which brings undesirable consequences for the inferential analysis (24). From the univariate regression (stepwise), the number of remaining variables to LST (n = 7), BMC (n = 5) and FM (n = 4) showed high r^2^_adj_ (0.90 to 0.94), while multicollinearity varied from low (L = 55.0) to moderate (L = 275.0) for the independent common variables for the three dependents variables (Table 2), a multivariable analysis.

**Table 2:** Univariate regression and multicollinearity for selecting common independent variables at least twice (bold)

Lean soft tissue		Bone mineral content			Fat mass	
*Variables*	*Eigenvalues*	*Variables*	*Eigenvalues1*	*Eigenvalues2*	*Variables*	*Eigenvalues*
Wt	0.255	Ht	0.275		**Wt**	0.110
SiSk	0.041	**Wt**	0.140	0.275	**HaSk**	0.027
HaSk	0.031	Um_Ab_	0.010	0.112	**CaCi**	0.006
WaCi	0.018	PHV	0.002	0.009	BiAc	0.002
TrSk	0.010	**SiSk**	0.001	0.001		
CaCi	0.002					
MiWa	0.002					
*r* ^*2*^_*adj*_ *= 0.946*	*L = 127.5*	*r* ^*2*^_***adj***_ *= 0.905*	*L = 275.0*	*L = 275.0*	*r* ^*2*^_***adj***_ *= 0.904*	*L = 55.0*

*Wt* weight, *Si* Supra-iliac, Sk Skinfold, *Ha* Horizontal abdominal, *Wa* Waist, *Ci* Circumference, *Tr* Triciptal, *Ca* Contracted armm, *MiWa* Middle waist, *Ht* Height, *Um*_*Ab*_ Umbilical abdominal, *PHV* years for peak height velocity, *BiAc* bi-acromial breadth, *r* ^*2*^_*adj*_ adjusted coefficient of determination, *L* multicollinearity

From the multivariate regression model, statistical significance (Pillai) of the F values (for < 2.2e^-16^ implies the notation < 2.2 x 10^-16^) was found for four predictor anthropometric variables.

The precision of the model was initially tested in univariate analysis (Enter method) while maintaining low multicollinearity and high correlation coefficients between measured and predicted values (Table 3). Multicollinearity was low, ranging from tolerance for eigenvalues (L<100), VIF (<10.0), and the determination of simple coefficients (r^2^) and adjusted (r^2^ adj).

**Table 3:** Parameters, precision and validity of multicomponent anthropometric model, to predict body composition in peripubertal girls

	β LST (kg)	β BMC (kg)	β FM (kg)	F num.	p (>F) *Pillai*	Sig.
Intercept	-1.8504187	0.373397388	2.8461916			
Weight	0.6662657	0.022218491	0.3645630	890.28	<2.2e^-16^	***
SiSk	-0.2157279	-0.010010969	0.1000325	54.97	<2.2e^-16^	***
HaSk	-0.2069373	-0.006453997	0.2888978	6.56	0.0005247	***
CaCi	0.3411678	0.008478514	-0.4752146	2.84	0.0435579	*
Precision						
r^2^	0.9392	0.8578	0.9056			
r^2^(adj)	0.9361	0.8506	0.9008			
SEE (kg)	2.0422	0.1012	2.2300			
Cross-validation				**(>) VIF**	**Eigenvalue**	
PRESS	329.300	0.809	392.852	7.282	89.000	
Q^2^_PRESS_	0.9272	0.8112	0.8872			
S_PRESS_	0.2718	0.0142	0.2966			

*LST* Lean soft tissue, *BMC* Bone mineral content, *FM* Fat mass, *Sig*. = 0,000 ‘***’; 0,001 ‘**’; 0,05 ‘*’, *SiSk* Supra-iliac skinfold, *HaSk* Horizontal abdominal skinfold, *CaCi* Contracted arm circumference, *r* ^*2*^ coefficient of determination (observed and cross-predicted), *r* ^*2*^ *(adj)* adjusted coefficient of determination, *SEE* residual standard error of estimate, *PRESS* sum of squares of residuals, *Q* ^*2*^_*PRESS*_ press coefficient of determination, *SEE*_*PRESS*_ press standard error of estimate, *VIF* Variance inflation factor, *Eigenvalue* characteristic values of regression

**Fig. 1:**
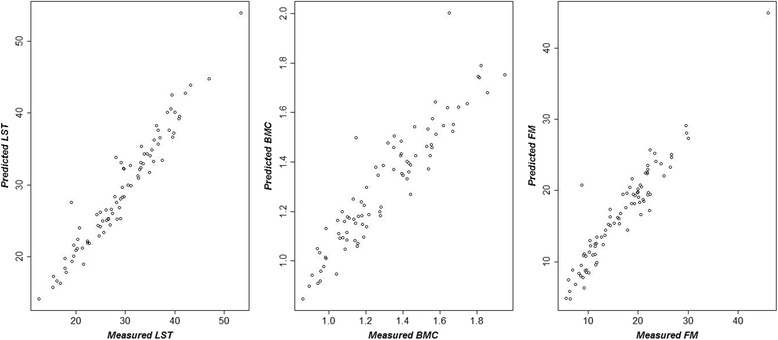
Dispersion between measured and predicted values of lean soft tissue (LST), bone mineral content (BMC) and fat mass (FM) of girls

The graphic expression of dispersion and the correlation between predicted and measured values is shown in Fig. 1.

The results of the residuals sum of squares (P_RESS_), the coefficient of determination PRESS (Q^2^_PRESS_) and the standard deviation of PRESS (S_PRESS_) obtained for each dependent variable, were also expressed (Table 3). These results suggested that the multivariate prediction model is valid for LST, BMC and FM since it indicates consistency with the conditions defined in the method for Q^2^_PRESS_ be close to "1" and S_PRESS_ close to "0". Thus, the final model for each dependent variable including Residual Tissue (RT) as the difference for the BW, can be stated as:

$$ {\displaystyle \begin{array}{c}\mathrm{LST}=0.6662657\ \mathrm{BW}\hbox{-} 0.2157279\ \mathrm{SiSk}\hbox{-} 0.2069373\ \mathrm{HaSk}+0.3411678\ \mathrm{CaCi}\hbox{-} 1.8504187\\ {}\mathrm{BMC}=0.0222185\ \mathrm{BW}\hbox{-} 0.1001097\ \mathrm{SiSk}\hbox{-} 0.0064539\ \mathrm{HaSk}\hbox{-} 0.0084785\ \mathrm{CaCi}+0.3733974\\ {}\mathrm{FM}=0.3645630\ \mathrm{BW}+0.1000325\ \mathrm{SiSk}\hbox{-} 0.2888978\ \mathrm{HaSk}\hbox{-} 0.4752146\ \mathrm{CaCi}+2.8461916\\ {}\mathrm{RT}=\mathrm{BW}\hbox{-} \left(\mathrm{LST}+\mathrm{BMC}+\mathrm{FM}\right)\end{array}} $$

*Where: LST = lean soft tissue; BMC = bone mineral content; FM = fat mass; RT = residual tissue; BW = body weight (kg); SiSk = supra-iliac skinfold (mm); HaSk = horizontal abdominal skinfold (mm); CaCi = contracted arm circumference (cm).*

## Discussion

As novel finding in this study, anthropometric models were developed and validated to determine FM, LST and BMC in a female pediatric sample, using DXA as the reference method. Adult models are not expected to describe or estimate body composition in children and adolescents [21]. Limitations in current available techniques to evaluate body composition hampers, in several circumstances, the measurement of the main body compartments in the molecular level [7]. In our study, DXA returned absolute measures of LST, BMC and FM in a 3-C approach, which represents estimation advantages by minimizing error in a single scan. However, DXA is almost restricted to the medical environment. Consequently, anthropometry is an alternative method since the anthropometric model was valid to estimate body composition using DXA as reference. Greater predictive accuracy was obtained through four anthropometric variables to simultaneously predict LST, BMC and FM.

DXA has great potential in pediatric studies, low level of ionizing radiation exposure, convenience and speed in the estimation of multicomponent body, which allows better interpretation of BC during growth and appropriate association with anthropometric estimates. Since the 80s, predictive equations of FM and FFM by anthropometry and BIA, using DXA (or other 3-C models) and 4-C as criterion methods [22], have been used, although none try to predict more than one component, otherwise only FM or FFM.

The known pediatric equations of Slaughter and colleagues used skinfolds models derived from a 4-C model [23]. However, lower coefficients of determination (r^2^ = 0.88) and greater standard errors of estimation (SEE = 3.80%) were found for FM determination, when compared to our results, (r^2^ > .94; SEE < 2.25%; S_PRESS_ = 0.01 to 0.30) and no cross-validation was provided.

In ages younger than 10 years, the dispersion of body tissues tends to be lower than in subsequent ages [19]. This implies that the pre pubertal growth and maturation are quite compatible. At this age, the change in hormonal concentration that occurs during sexual maturation has not yet started [12]. The density of FFM is influenced in large part by bone mineral because the density of bone is markedly higher than that of other components of the FFM. During this special period of life there is an unstable relationship between BMC and muscle mass in comparison to adulthood [24]. In addition, the increase of other body components during growth [25] is markedly higher in FM until about 13 years, registering increases from childhood to adulthood of 17% in girls and 15% in boys. When presenting exponential reduction, no significant changes are found for the three components after age 20 in girls and age 21-22 in boys [26].

Besides extending to girls with high body mass, this new methodology overcome traditional limitations of bi-compartmental models by offering an alternative multivariate analysis, a new way for a comprehensive approach [7, 21, 27, 28]. The observed high coefficients values and low errors estimation (Table 3) reflected a usual good validity when using our laboratory proposed equations [2]. Several factors can affect the accuracy of a predictive equation as: the dependent variable validity; accuracy of predictive variables; statistical intra and inter-biological relationship between independent and dependent variables; appropriated use of statistical methods to formulate the equation; and adequate sample size [29]. The necessary conditions for the proposition of a predictive model must meet statistical requirements for a safe and reliable model/equation.

Assumptions for good predictive models of body composition recommend that models should be proposed for a wider clinical application [30], make valid assumptions in relations between the components [28], i.e., if relations between FFM, FM and TBW are constant for all ages. Some equations proposed are more well-founded descriptions and specific to predict only one component [31], or restricted age range [32]. When it involves wider range age (5-19 years), adopt sophisticated laboratory techniques and expensive procedures [33]. Furthermore, the inclusion of independent variables with strong statistical and biological relationship with the variable of interest, certainly increases its predictive power [28]. The internal validation method PRESS [34], as in our equations, confirms the effectiveness of the model in predicting the body components with high internal validity, high coefficients of determination (Q^2^_PRESS_ = 0.81 a 0.93) and low prediction errors (S_PRESS_ = 0.01 a 0.30).

The anthropometric measurements are obtained easily and at a low cost while preserving the accuracy and low error to be considered as an alternative to DXA [35]. We recognize as limitation of this study that DXA is not a gold standard for pediatric body composition models. But conversely, the four compartments (4-C models) is the current state of the art method since it considers the variability of the main FFM components [36]. However, it is also subject to propagation of measurement errors, is time consuming, and requires sophisticated and highly specialized technical equipment. Moreover, it is restricted to research facilities and has high costs, making it difficult to use in large samples [37]. Nevertheless, DXA has been used by various researchers to develop pediatric equations [19, 38–41], therefore considered as a valid and reliable method [42]. Other limitation consider that the accurate measurement of LST, especially in athletes, requires strict observation of hydration, which may affect DXA assessment [43]; as well the menstrual cycle of girls [44], not controlled in this study. However, the girls in the study had a low level of maturation (PHV = -3.16) and were not athletes, minimizing possible errors of analysis.

Additionally, nutritional data, social or economic status, hereditary or level of sports training were not controlled in this study and may interfere with the female body composition. The small sample size may compromise the accuracy of the models since the sample may not have a well balance representation of age and adiposity levels. In addition, ethnic differences in this sample, although comprising a highly mixed-race, may also limit the generalization of these equations for other populations as the number of females in each ethnic group was not well balanced [2]. Although racial and sports practice showed no interactions terms with anthropometric variables in explaining the reference variables, research is required to test the accuracy of the proposed model for other ethnic groups. Finally, for longitudinal body composition assessment, the accuracy of these models requires further validation, or estimates for age groups.

## Conclusions

The goal of this study to develop and validate an anthropometric predictive model to simultaneously estimate LST, BMC and FM was achieved. These models provide a practical, low cost, and reliable tool in assessing body composition of a specific female pediatric population. These models can be used to determine and monitor body growth in peripubertal girls, either to assess health indicators (i.e., overweight control, obesity and the associated risks), or improve sports performance (i.e., body adequacy and physical preparation for sports). However it should be tested and validated for other ethnic groups.

The internal validation method PRESS [34] confirms the effectiveness of the model in predicting the body components with high internal validity, with high coefficients of determination and low prediction errors. These models may be a valid alternative to estimate body composition in girls and allow application in clinical environment or field settings.

## Abbreviations

*Z*_*y*_: degree of confidence
*ε*: maximum error
*σ*^2^: variance
2-C: two-compartment model
A: age (years)
BMC: Bone mineral content
BMI: Body mass index
BW: Body weight
CaCi: Contracted arm circumference
DXA: Dual energy X-ray absorptiometry
FFM: Fat-free mass
FM: Fat mass
HaSk: Horizontal abdominal skinfold
Ht: Height
L: multicollinearity
Lh: Legs height
LST: Lean soft tissue
PHV: Peak height velocity
PRESS: sum of squares of residuals
Q^2^_PRESS_: PRESS coefficients of determination
r: coefficients of correlation
SEE: Standard errors of estimation
Sh: Seating height
SiSk: Supra-iliac skinfold
S_PRESS_: PRESS error
TBW: Total body water
TEM%: relative technical error of measurement
TEM: absolute technical error of measurement
VIF: Variance inflation factor
Wt: body mass (kg)
β: beta parameter
